# An anthropomorphic beating heart phantom for cardiac X‐ray CT imaging evaluation

**DOI:** 10.1120/jacmp.v11i1.3129

**Published:** 2010-01-28

**Authors:** Thomas Boltz, William Pavlicek, Robert Paden, Markus Renno, Angela Jensen, Metin Akay

**Affiliations:** ^1^ Diagnostic Imaging Physics Mayo Clinic Scottsdale Scottsdale AZ USA

**Keywords:** heart phantom, cardiac, CT, Anthropomorphic, image quality

## Abstract

The current work describes an anthropomorphic beating heart phantom constructed as a tool for the assessment of technological advances in cardiac X‐ray computed tomography (CT). The phantom is comprised of a thorax, a compressor system, an ECG system, a beating heart with tortuous coronary arteries, and the option to add or remove pathologies such as aberrant beats, stents, and plaques. Initial trials with the phantom have shown its utility to assess temporal resolution, spatial resolution, radiation dose, iodine contrast, stents, and plaques.

PACS numbers: 87.59.Fm, 87.57.Ce

## I. INTRODUCTION

X‐ray coronary computed tomography angiography (CCTA) presents challenges in obtaining images of high quality.^(1)^ Patients commonly receive beta blockers and contrast injection while being instructed to hold their breath for 15 to 20 seconds. The acquisition is timed with the administration of contrast material in consideration of the patient holding deep inspiration, using ECG monitoring of the heart rate to determine the appropriate scan selections at the operator console.^(2)^ The CT scanner acquires data using continuous irradiation at reduced pitch (<0.3) or using fixed irradiation pulse lengths that match quiescence in the ECG.^(3)^ Patients undergoing a CCTA have an X‐ray acquisition prescribed that is based upon their individual body habitus, heart rate, and plaque or stent presence.^(4)^

The intent of this work is to fabricate a repeatable cardiac imaging test that can facilitate identification of image acquisition protocols to accommodate a given patient's complexities.^(^ ^5^ ^,^ ^6^ ^)^ A beating heart phantom is described that fully simulates the task of imaging of the heart with CT, including several of the variables described above.

## II. MATERIALS AND METHODS

### A. Average patient specifications

**Figure 1:**
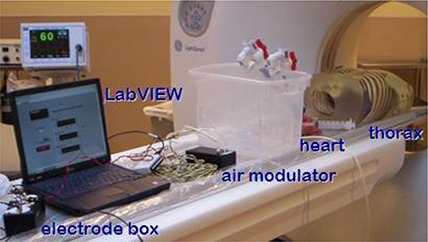
The beating heart phantom is shown in a CT scanner. The thorax is far right, near the gantry, and the heart is to the left of the thorax with tubes connected for fluid and air exchange. Below the heart and outside of the picture is the air compressor. The air modulator is the first black box to the left of the heart, while LabVIEW is displayed on the laptop and the ECG electrode connection is the black box on the left of the laptop. The ECG monitor is seen in the background with a 60 bpm ECG waveform that the phantom is simulating.

The physical attributes of the beating heart phantom were based upon a review of X‐ray CT examinations of 200 patients having received a CCTA using either a Siemens Somatom Sensation 64 CT scanner or a General Electric LightSpeed VCT 64 CT scanner. To assess thorax characteristics surrounding the heart, measurements were taken of the lateral width and the anterior to posterior length. The Hounsfield unit (HU) values within the lungs, soft tissue, and bone were measured and recorded. To assess heart chamber characteristics, measurements included the longest distance from valve to apex, anterior to posterior, and left to right dimensions within the heart. The myocardium was then measured for HU values, as was the blood within the ventricles and the mixture of blood and contrast material present following intravenous contrast injection. Inner diameters of the coronary arteries were also measured within image space. The left anterior descending (LAD), left circumflex (LCX), and the right coronary artery (RCA) were all measured at the proximal, medial, and distal segments. The resulting beating heart phantom includes multiple components fabricated to match the average patient values. These components are shown in Fig. 1.

### B. The thorax

The thorax phantom is intended to house the beating heart and to produce scatter as exhibited in actual patients. The phantom's thorax is fabricated with ribs, sternum, spine, soft tissue, and lung mimicking materials with densities and HU values matching human bone, soft tissue, and lung tissues. CT scanners that modulate the current (mA) exposure simultaneously with the X‐ray triggering to the ECG may suffer from higher noise when the quiescent time of the heart occurs during a time when X‐ray projections in the lateral direction are collected. Evaluation of CCTA image quality under these circumstances is preferable. A cavity for heart placement was constructed in the thorax, allowing the phantom heart position to match the heart position seen in the patient population. An air compressor system causes the heart to beat within the thorax. The compressor is connected to the heart with lengths of plastic tubing so that it can be located in the scan room or at the operator console.

### C. The compressor system

The compressor system has four components: the compressor, the air modulator, the tubing throughout the ventricular walls of the heart, and the LabVIEW signals.^(7)^ Pneumatic tubing connects the air compressor, the air modulator, and ventricular wall tubing in series. Signals from LabVIEW to the air modulator tell the air modulator when to initiate inflow and outflow of air through the ventricular tubing. The atria and ventricle chambers are separate compartments from the pneumatic tubing that runs throughout the ventricular walls of the heart. The separate chamber and tubing compartments allow contrast medium to fill the chambers themselves while air moves in and out of the ventricular walls. Outflow of air from the ventricular tubing constricts the ventricular walls and causes the heart to contract. Inflow of air through the ventricular tubing expands the ventricular walls and causes the heart to relax. In a sequential pattern, the increase and decrease of air pressure within the ventricular tubing causes contraction and relaxation of the heart's ventricles.

### D. The ECG system

**Figure 2:**
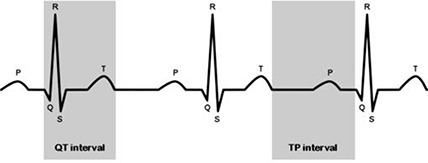
Ventricular systole occurs during the QT interval of electrical activity. The left and right ventricles of the heart contract during this time. It is a period of high movement and visualization of the coronary arteries is more difficult than during ventricular diastole. During the TP interval, the heart relaxes while it fills with circulating blood. Phases within 70% to 80% of the R‐to‐R interval occur during the TP interval when minimum coronary motion is expected.

Simulating a CCTA exam requires an ECG trace for both gating the X‐ray exposure on the scanner (i.e. mimicking the electrical activity of the patient) and causing the proper cardiac motion in the beating heart. An imaging protocol that needs to trigger a momentary exposure during minimal heart motion attempts to time the X‐ray exposure during the TP interval. The phantom's ECG trace allows for gating and represents true phantom motion during the QT interval and phantom quiescence during the TP interval. Figure 2 outlines the phases of the human ECG signal as described.

LabVIEW, a software program capable of communicating with multiple hardware devices, is utilized to meet the several demands upon the ECG trace. The compressor's trigger signal and the scanner's gating signal both originate from LabVIEW and synchronize phantom motion to the QT interval registered on the scanner. Initial scanning with the LabVIEW program revealed that the phantom's ECG trace registered a QT interval on the scanner one half second before the compressor signal could initiate the associated movement in the heart. The ECG signal was modified to permit a timing offset in increments of one hundredth of a second and to facilitate matching.

### E. The heart

The phantom's heart chambers and coronaries can be sealed for use with contrast medium. The beating ventricles force fluid in and out of the atria and induce movement similar to atrial diastolic filling and systolic ejection. The left atrium and left ventricle allow fluid to flow from one chamber to the other. The right atrium and right ventricle allow fluid to flow from one to the other and into the aorta and coronary arteries. When the ventricles contract, fluid pushes out of the ventricles and into the atria and coronary arteries. Ventricular contraction and relaxation displace the coronaries by about one diameter, which is the distance found with actual patient measurements. Once placed within the thorax, the contrast‐filled beating heart phantom exhibits motion similar to that of a patient.^(8)^

### F. Phantom pathology

Initial phantom trials involved the assessment of temporal resolution. Qualitative analysis of the phantom's motion artifacts provided insight into the temporal resolution achieved on a given CT scanner. Visual image comparisons were made between scans when the phantom was physically not beating and scans when the phantom was beating but imaged within the TP interval. Motion artifacts would be identified when viewing the two image sets. Ideally, little to no difference with images acquired of a stationary phantom would be identified.^(9)^

In order to evaluate a scanner's ability to perform multiple phase reconstructions (using more than one cardiac cycle to capture all the projections needed to form one image of one slice of anatomy of the heart), aberrant beats can be simulated in the ECG signal. In normal ECG signal production, the electrode box generates a changing voltage per second in the form of a sawtooth wave. The peaks in the sawtooth wave trigger a QT interval in the ECG monitor, achieving a steady ECG signal. To generate aberrant beats, a second wave was added to the ECG signal that changes voltage more slowly over time, resulting in an irregular peak every 7 seconds. These beats create motion and irregular ECG data during a phase of data collection, providing a means for postprocessing evaluation.

Coronary plaques were designed in a 3D CAD environment called SolidWorks and custom machined to fit within the coronary arteries of the heart phantom.^(10)^ Lipid tissue density plaques, lipid‐fibrous density plaques, and fibrous plaques were designed for the phantom's LAD, LCX, and RCA vessels. Each plaque matches the inner diameter of a specific coronary and creates a specific blockage to that artery. The known diameters of the plaques can be compared to diameters measured in image space. Spatial resolution assessment can then be inferred from diameter comparisons that involve 0.4 mm and lower diameters. After developing a deployment method for the plaques, the method was used for placing and removing stents into the coronary arteries, thus allowing visualization of a range of stents imaged using the beating heart phantom. Direct comparisons of image quality between CCTA and conventional X‐ray cardiac catheterization are also possible. This is important for evaluation of stent damage or possible re‐stenosis.

### G. Phantom scanning

Initial use of the phantom occurred using the five cardiac CT protocols listed in Table 1. The HU specifications of the phantom were measured with Retrospective Protocol 1. Gating capabilities were validated for the three CT scanners reported in Retrospective Protocols 1, 2, and 3. Aberrant beat and temporal resolution studies were performed with Retrospective Protocol 3. Retrospective versus prospective gating was evaluated with Retrospective Protocol 1 and Prospective Protocol. A high‐resolution technique was evaluated with the High Resolution Protocol.

**Table 1:** Scan protocols.

*Parameters*	*Retrospective 1*	*Retrospective 2*	*Retrospective 3*	*Prospective*	*High Resolution*
Manufacturer	GE	GE	Siemens	GE	GE
Model	LightSpeed VCT 64	Discover HD CT750 64	Somatom Sensation 64	LightSpeed VCT 64	Discovery HD CT750 64
Scan Name	Snapshot Segment	Snapshot Segment	ThorCardioECG 033s	Snapshot Pulse	Snapshot Pulse High Res
Acquisition	Helical	Helical	Helical	Axial	Axial
Data Channels	64 by 0.625 mm	64 by 0.625 mm	32 by 0.6 mm	64 by 0.625 mm	64 by 0.625 mm
Pitch	0.22	0.22	0.3	n/a	n/a
Rotation	0.35 sec	0.35 sec	0.33 sec	0.35 sec	0.35 sec
Exposure/Rotation	0.35 sec	0.35 sec	0.33 sec	0.23 sec	0.23 sec
Voltage	120 kVp	120 kVp	120 kVp	120 kVp	120 kVp
z‐Sampling	routine	routine	double	routine	routine
x,y‐Sampling	routine	routine	double	routine	double
Current	750 mA	750 mA	720 mA	750 mA	750 mA
Current Modulation	OFF	OFF	OFF	OFF	OFF
Padding	n/a	n/a	n/a	0 sec	0 sec
Scan FOV	50 cm	50 cm	50 cm	50 cm	50 cm
Recon FOV	25 cm	25 cm	25 cm	25 cm	25 cm
Recon Filter	Standard	Standard	B20f	Standard	HD Standard
Recon Slick Thick	0.625 mm	0.625 mm	0.75 mm	0.625 mm	0.625 mm
Recon Increment	0.625 mm	0.625 mm	0.7 mm	0.625 mm	0.625 mm
Recon ECG Phase^a^	75%	75%	65%	75%	75%
CTDI Vol.	52 mGy	52 mGy	43 mGy	8 mGy	8 mGy
Effective Dose	21 mSv	21 mSv	17.4 mSv	2.4 mSv	2.4 mSv

^a^ The 65% phase on Siemens is equivalent to the 75% phase on GE because the 65% is measured from the top of the peak in the QT interval and the 75% is measured from the start of the peak in the QT interval. Both phases represent the TP interval of quiescence in the ECG.

Further studies, not reported here, have been initiated to more fully assess CCTA with the phantom. One ongoing assessment is radiation dose and image quality comparisons with multiple cardiac protocols on a single CT scanner, and between similar technologies on separate CT scanners. Another planned assessment is an evaluation of spatial resolution on multiple CT scanners during CCTA.

## III. RESULTS & DISCUSSION

### A. Phantom specifications

**Figure 3:**
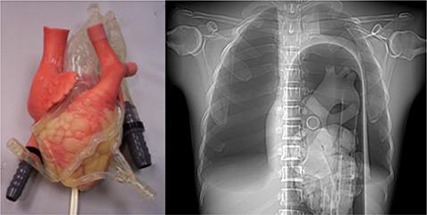
Anterior view of the heart phantom (left); an AP radiographic image of the heart phantom (right) within the thorax, showing the scatter environment that houses the heart.

The functional beating heart phantom can be seen in Fig. 3. It is complete with soft silicone chambers, soft silicone coronary arteries, and tissue densities that cause X‐ray scatter similar to an average patient.^(11)^

Ports for accessing the heart chambers through the inferior vena cava and the aorta are shown, as well as ports for filling the coronaries through their most distal portion. These arteries may appear large, but the inner diameters match those of an average patient. Phantom specifications were measured with the Retrospective Protocol 1 acquisition and compared with the CCTA patient population. Patient averages and the phantom specifications can be seen in Table 2.

**Table 2:** Average patient measurements compared with phantom specifications.

	*Average Patient*	*Phantom*
Thorax anterior posterior	211 mm±25	209 mm
Thorax left to right	285 mm±29	302 mm
Thorax soft tissue	44 HU±18	25 HU
Thorax bone tissue	708 HU±115	751 HU
Thorax lung tissue	−778 HU±57	−684HU
LV Aortic valve to apex^a^	133 mm±15	77 mm
LV anterior posterior	110 mm±16	67 mm
LV left to right	112 mm±11	93 mm
LV myocardium	115 HU±24	150 HU
LV blood	41 HU±5	20 HU
LV blood and contrast	34 2HU±89	30 HU–1,000 HU
LAD proximal	4 mm±0.5	3.7 mm
LAD medial	3.5 mm±0.3	3.0 mm
LAD distal	1.8 mm±0.2	2.1 mm
LCX proximal	3.0 mm±0.6	3.2 mm
LCX medial	2.0 mm±0.4	2.4 mm
LCX distal	1.5 mm±0.4	1.8 mm
RCA proximal	4.0 mm±0.6	3.9 mm
RCA medial	3.5 mm±0.4	3.4 mm
RCA distal	2.0 mm±0.2	1.7 mm
Heart Rate	55–75 bpm^b^	40–85 bpm

^a^ LV=Left Ventricle

^b^ bpm=beats per minute.

Hounsfield unit measurements for the patient population and the phantom specifications were taken from images acquired using 120 kVp. The HU values for saline simulating blood in the phantom were measured within the chambers. Phantom densities are generally higher than the reported average patient densities, but the contrast ratio between the vessels and the contrast medium can be duplicated to make image quality evaluations applicable.^(12)^ The measurements for the left ventricle are provided as an example of the measurements performed for all chambers.

### B. ECG gating

**Figure 4:**
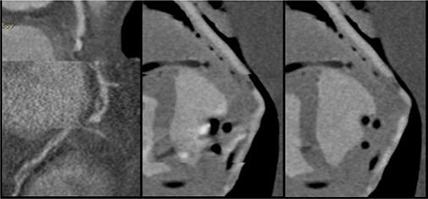
Patient RCA (left) reconstructed with 3 phases: TP interval on the top, QT interval in the middle, and TP interval at the bottom; the phantom RCA (middle) reconstructed with 3 phases: TP interval on the top, QT interval in the middle, and TP interval at the bottom; the phantom RCA (right) reconstructed with only the QT interval.

After achieving accurate propagation of the ECG and compressor signals, typical patient heart rates were tested with routine scanning protocols. Comparison of beating and not beating phantom scan reconstructions were made to determine the level of cardiac motion artifact relative to motion artifact found in patients. Figure 4 shows a comparison of a patient's RCA movement with that of the beating phantom's RCA movement, using the Retrospective Protocol 3.

As seen in Fig. 4, no appearance of motion was observed for Retrospective Protocols 1, 2, and 3 from Table 1. For heart rates ranging from 40 bpm to 85 bpm, motion artifact is not present during the TP interval of the ECG.

The completion of the ECG gating validation was followed by a study between retrospective versus prospective gating. Retrospective techniques, using pitch less than 0.3, continuously collect data from several heart beats during all heart cycle phases. This technique ensures complete coverage of the heart and allows for correction of motion artifacts caused by non‐steady cardiac rhythms. Prospective techniques only irradiate during the predicted TP interval, implying cardiac quiescence. This step‐and‐shoot mode reduces dose, but limits the ability to correct motion artifacts due to the smaller number of cardiac phases acquired. Once the beating heart phantom had been scanned with the Retrospective Protocol 1 and Prospective Protocol from Table 1, it was evident that image quality could be maintained with either technique. It was also confirmed that the retrospective technique delivered 21 mSv effective dose, while the prospective technique delivered only 2.4 mSv effective dose. The phantom study proved that prospective gating could be prescribed for patients with heart rates up to 75 bpm, providing that the heart rate is steady (because the phantom's heart rate was steady during testing).

### C. Phantom pathology

Scanning the phantom with the aberrant beat signals simulates motion artifacts that the scanner and post processing should be able to eliminate given multiple phases. The phantom images with motion artifact in a phase reconstruction can be postprocessed to evaluate beat rejection software from a vendor. Alternatively, the beat rejection software during acquisition can be compared.

**Figure 5:**
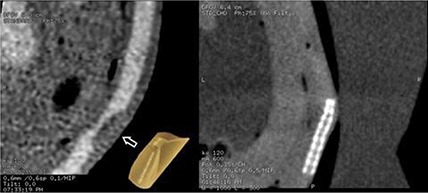
A curved center‐seeking multiplanar reformat (CSCMPR) (left) of the phantom's LCX and a picture of a machined fibrous plaque, with arrow denoting the location of the machined fibrous plaque within the scanned artery; CSCMPR of the phantom's RCA (right) from a high‐resolution scan with a stent in the lower region of the artery.

Removable coronary plaques provide a test of iodine contrast enhancement and spatial resolution. The plaques themselves can be scanned free in air, immersed in saline, or immersed in contrast medium. The effects of increased contrast on perceived plaque HU can then be assessed as a function of scatter environment and/or kVp.^(13)^ Such assessments would indicate the protocol that properly visualizes the known size and shape of the plaque. Plaque phantom stenoses of 40%, 60%, and 80% were all fabricated for each coronary segment and each density type. Further plaque assessment would include evaluating the accuracy of stenoses within the image data as compared to known stenoses. Figure 5 depicts the presence of plaque phantoms and stents within the beating heart phantom.

In the left image of Fig. 5, an arrow indicates the presence of a fibrous tissue density plaque insert in the LCX. This fibrous plaque has been machined to create a 60% blockage in the medial portion of the LCX. Image based measurements of the blockage estimate the blockage to be 68%.^(14)^ As for the stent in the right image of Fig. 5, the phantom results show that a stent can be visualized with the High Resolution Protocol from Table 1 while beating 63 bpm. The High Resolution Protocol seen in Fig. 5 utilized a double sampling in the x–y plane and delivered 2.4 mSv effective dose, which is the same dose that a routine prospective exam would deliver. When blooming is present, the detected signal is so strong that the stent appears to be bright around its entire perimeter and limits visualization of possible tissue between struts. The phantom provides a means of evaluating the level to which a CT scanner visualizes re‐stenosis and reduces blooming.

## IV. CONCLUSIONS

A beating heart phantom was constructed that reasonably duplicates an average patient's cardiac exam in terms of scatter, cardiac tissue densities, coronary size, motion, heart rate, and contrast material. Representative pathology can be simulated such as anomalous ECG activity, and stents and plaques located in the proximal, medial, and distal segments of the coronary tree. The phantom offers the ability to isolate X‐ray CT acquisition parameters and provides direction for optimal imaging strategies including: radiation dose, temporal resolution, spatial resolution, iodine image contrast, image reconstruction parameters, and accuracy of stenosis and plaque composition.

## ACKNOWLEDGEMENTS

The authors would like to thank Mayo Clinic Arizona and Arizona State University for providing the funds to build the beating heart phantom.
